# Doubling Throughput of a Real-Time PCR

**DOI:** 10.1038/srep12595

**Published:** 2015-07-27

**Authors:** Christian D. Ahrberg, Pavel Neužil

**Affiliations:** 1 Kist-Europe, Saarbrücken, Saarland, 66123, Germany; 2 Central European Institute of Technology, Brno University of Technology, Technická 3058/10, CZ-616 00 Brno, Czech Republic; 3 Northwestern Polytechnical University, School of Mechanical Engineering, 127 West Youyi Road, Xi’an, Shaanxi, 710072, P.R.China

## Abstract

The invention of polymerase chain reaction (PCR) in 1983 revolutionized many areas of science, due to its ability to multiply a number of copies of DNA sequences (known as amplicons). Here we report on a method to double the throughput of quantitative PCR which could be especially useful for PCR-based mass screening. We concurrently amplified two target genes using only single fluorescent dye. A FAM probe labelled olionucleotide was attached to a quencher for one amplicon while the second one was without a probe. The PCR was performed in the presence of the intercalating dye SYBR Green I. We collected the fluorescence amplitude at two points per PCR cycle, at the denaturation and extension steps. The signal at denaturation is related only to the amplicon with the FAM probe while the amplitude at the extension contained information from both amplicons. We thus detected two genes within the same well using a single fluorescent channel. Any commercial real-time PCR systems can use this method doubling the number of detected genes. The method can be used for absolute quantification of DNA using a known concentration of housekeeping gene at one fluorescent channel.

The first demonstration of polymerase chain reaction (PCR) in 1983 is considered one of the greatest scientific achievements of the 20^th^ century^1^. It revolutionized many areas of science, due to its ability to multiply a number of copies of DNA sequences (known as amplicons), using either DNA directly or complementary DNA after reverse transcription from RNA. The PCR cocktail called master mix contains free nucleotides, sets of primers and other compounds, besides the polymerase enzyme. The primers are short DNA sequences, one complementary to the DNA strand of interest in a forward direction and the second one complementary in reverse. The classic PCR method required the employment of a post-processing step such as electrophoresis or hybridization to verify the presence and purity of an amplicon.

A few years after the first PCR demonstration, a fluorescent marker was added to the PCR mixture to monitor the reaction in real time^2^. It also allows the determination of an initial DNA concentration. Thus, this method is often called a quantitative PCR (qPCR). The most popular qPCR is based on intercalating dyes, such as SYBR Green I. The dye produces its fluorescence only in the presence of double-stranded DNA. Thus, researchers can monitor the concentration of amplicons. Another outcome of this method is the melting curve analysis (MCA), typically conducted once the PCR is completed. The sample is slowly warmed up while its fluorescence is monitored. At elevated temperatures, the double stranded DNA amplicons start to convert to single stranded (melt) and consequently the amplitude of the fluorescence drops. When half of the DNA melts from double stranded into single stranded, the temperature is called the melting temperature (T_M_). It is characteristic of the DNA amplicon length and its sequence. The intercalating dye-based methods are frequently used as they are not specific to any particular DNA sequence.

Another popular qPCR method is based on a probe such as 6-carboxy-fluorescein (FAM)^3^. This probe has to be chemically bound to the oligonucleotides next to a quencher such as TAMRA or a black hole quencher. This PCR method is often called TaqMan or TaqMan chemistry^4^. Once the primers bind to the DNA template, the quencher typically gets separated from the fluorophore and its fluorescence amplitude increases. This method is gene specific but requires oligonucleotides to be synthesised with attached dye and a quencher. Fluorescence resonance energy transfer (FRET)-based methods are also popular^5^.

A method to detect more than one target sequence in a single sample is called multiplexing. It offers significant advantages compared to single PCR, such as more information per reaction and therefore time saving. Applications for multiplex PCR can be found in food sciences^6^, agricultural sciences^7^ or in human medicine. In human medicine, the method is often used to determine ratios of housekeeping genes for normalization^8^, assessing viral loads^9^ or determining the species^10^ or subspecies in bacterial^11^ and viral infections^12^. Researchers used end-point multiplex detection to identify different amplicons by the differences in their melting temperature by MCA^13^ in the presence of an intercalating dye^14^. This method requires amplicons with different melting temperature caused by different DNA lengths and sequences^15^. Alternatively, a gel electrophoresis can be performed after PCR to separate and detect the different DNA amplicons^16^. This method requires amplicons with different number of base pairs (length), resulting in different electrophoretic mobilities for each amplicon. Both methods are based on post-processing after PCR. Thus, they provide qualitative information as to whether the DNA sequence of interest was present or absent in the original sample. There is limited knowledge obtained about the quantity of DNA templates in the original sample.

The probe-based methods belong to the real-time PCR family. The results give quantitative information, thus also called quantitative PCR (qPCR). The most common approach is performing PCR with oligonucleotides attached to probes with different emission wavelengths and detect them using several fluorescence channels^17^. The number of genes identifiable per fluorescence channel can be further increased by creating colour codes^18^ for the different genes and extracting information on amplicon concentrations via linear combinations on the individual fluorescence channels^19^. Theoretically, the number of genes concurrently detectable is unrestricted, but there are various limits imposed through the optical filter system required and through the dye assays. Selecting an appropriate probe for the assay also becomes increasingly difficult and assay costs increase with the number of used probes.

What will happen if TaqMan probes, such as FAM, and intercalating dyes, like SYBR Green I, are combined in the same experiment? One of the earliest work reports combination of TaqMan probe with emission in the FAM spectrum and the asymmetric cyanine dye BOXTO (TATAA)^20^. BOXTO has an emission maximum at 552 nm, thus requiring a different filter set from FAM to be detected. In their work, they combined the dyes to quantify and detect the gene of interest using the TaqMan dye. Once the PCR was completed, they performed melting curve analysis (MCA) to detect nonspecific product formation by monitoring the fluorescence signal from BOXTO.

A few years later, PCR was performed with a primer set labeled with TaqMan probes6-Carboxyl-X-Rhodamine (ROX) or diSulfo - Cy5 carboxylic acid (Cy5) in presence of SYBR Green I intercalating dye to detect polymorphism in *Mycobacterium (M) tuberculosis* culture^21^. Both those dyes have different fluorescent distinctively different excitation/emission wavelength from SYBR Green I thus different filters have to be used for detection of each dye. With this combination of dyes and using primer sets for two different amplicons, the authors got three possible Boolean outcomes after completing the PCR. They either got a signal from the TaqMan probe and the intercalating dye, a signal from the intercalating dye or no signal. In the first case, they could conclude that M. tuberculosis was present and the polymorphism had not occurred. In the second case, M. tuberculosis was present and the polymorphism did occurred and in the last case either M. tuberculosis was not present or the PCR failed. The Boolean approach described by authors allows specifying which of the two phenotypes is present, as long as there is no mixture of the two phenotypes. Both works^20^,^21^ used different fluorescent channels to detect the probe and to detect the intercalating dye.

Recently, it was shown that two dyes with the same emission wavelengths could be combined^22^. Researchers used four different TaqMan probes labelled with FAM dye, phosphoramidite (HEX), Texas Red and Cy5 dyes combined with the intercalating dye SYBR Green I, which exhibits nearly identical excitation/emission spectra as FAM. They extracted quantitative data during the PCR from individual signals from TaqMan probes above the melting temperature of the amplicons to eliminate signal from the intercalating dye. Once the PCR was completed the MCA was performed to detect the unspecific amplification due to possible primer interactions.

In our approach, we take this idea one step further by measuring both the signal of the TaqMan probe as well as the combined signal of the TaqMan probe and intercalating dye during thermal cycling. This allows us to detect and quantify two different genes in the FAM fluorescent channel. We used primer sets for two different genes, a FAM based probe complementary to only one of the genes and SYBR Green I as an intercalating dye.

Both genes contributed to the total fluorescence amplitude in the presence of an intercalating dye such as SYBR Green I, as long as the temperature of the mixture was below the T_M_. The FAM-labelled probe separated from the quencher also contributed to the total fluorescence. Once the temperature was increased above the T_M,_ only FAM-based fluorescence contributed to the total fluorescence amplitude. We could then extract two amplification curves: the probe-based amplicon at the denaturation temperature (here called a denaturation curve) and the second one at the end of the extension step (here called an extension curve). This second amplification curve contains both DNA templates together. The concentration of the probe-linked amplicon is already known and can be subtracted from the extension curve, resulting in the concentration of the second amplicon. We can then determine the threshold cycle (C_T_) and via comparison with the PCR standard curve also the corresponding original template concentration of both DNAs. The major advantage of this method is the ability of simultaneous, quantitative detection of two DNA templates, using only a single fluorescent channel.

There are two mechanisms of fluorescence contributing to the extension curve: intercalating dye and the probe. The amplitude of this composite fluorescent signal always has to be higher than the one originating only from TaqMan probe extracted at the denaturation curve. Another reason why the signal originated from TaqMan probe is lower is its sensitivity to temperature. Its amplitude is inversely proportional to temperature and the denaturation curve is taken at 93 °C–95 °C, while the extension curve is captured at about 60 °C.

The method described above works even in extreme cases with one DNA template having significantly higher number of copies than the second one. Once the number of copies of the DNA linked to TaqMan probe is dominating the *C*_*T*_ value extracted from denaturation curve will be slightly higher than the *C*_*T*_ value extracted from extension curve. In the opposite case with non-TaqMan template number of DNA copies is dominating, the *C*_*T*_ extracted from the extension curve will be significantly lower than the corresponding *C*_*T*_ extracted from the denaturation curve. In both cases, the difference in both *C*_*T*_ determined the number of copies of the second DNA template.

We performed two sets of experiments. First, we used two common housekeeping genes: Glyceraldehyde 3-phosphate dehydrogenase (GAPDH) and hypoxanthine-guanine phosphoribosyltransferase (HPRT). The results could be used to calculate a normalization factor by method shown earlier^8^,^23^,^24^,^25^.

In the second experiment, we determined a viral load in the sample of haemagglutinin (HA) with both HPRT and GAPDH as housekeeping genes^26^.

## Material and Methods

### Experiments

Synthetic DNA templates for GAPDH, HPRT and HA (ATG Biosynthetics) were used for the experiments. The primers were designed using Roche Universal Probe Library Assay Design Center and purchased from Eurofins MWG Operon (Table 1). Universal probe #73 (Roche Molecular Systems) was used as TaqMan probe corresponding to HPRT together with the Roche LightCycler TaqMan Master Mix. The reaction mixture consisted of 4 μL master mix, 1 μL of SYBR Green I 10,000 x solution (Lonza) diluted by a factor of 500. The primers and probe were added with concentrations of 1.8 μM and 200 nM, respectively. A sample template was then pipetted in and the mixture volume was increased up to 20 μL by adding de-ionized water produced by a Milli-Q ProgradT3 (Millipore) column after autoclaving. Data collection and thermal cycling were done by Roche LightCycler Carousel-Based system using a temperature profile according to the master mix specifications. We used continuous fluorescence measurement mode to capture the data. The measurement can be simplified by collecting fluorescence amplitude data only at the end of the extension and denaturation step with the same quality of results.

Standard curves were recorded in two sets of experiments. In the first set, the concentration of the TaqMan gene was constant while the concentration of non-TaqMan gene was varied (Tables 2 and 3). In the second experiment set concentration of non-TaqMan gene was constant while the TaqMan gene concentration was varied (Tables 4 and 5).

Lastly, an experiment was conducted to determine the concentration of both genes in the presence of a third one. The sample contained HPRT with concentrations of 3.1 × 10^−7^ ng/μl, GAPDH with a concentration of 3.1 × 10^−6^ ng/μl and HA with a concentration of 6.25 × 10^−6^ ng/μl. We only used two primers at a time, either HPRT and GAPDH primers or HPRT and HA primers. A second sample with the concentrations of 1.6 × 10^−7^ ng/μl HPRT, 6.25 × 10^−6^ ng/μl GAPDH and 1.6 × 10^−5^ ng/μl HA was also tested in the same manner.

### Data Analysis

**Figure 1:**
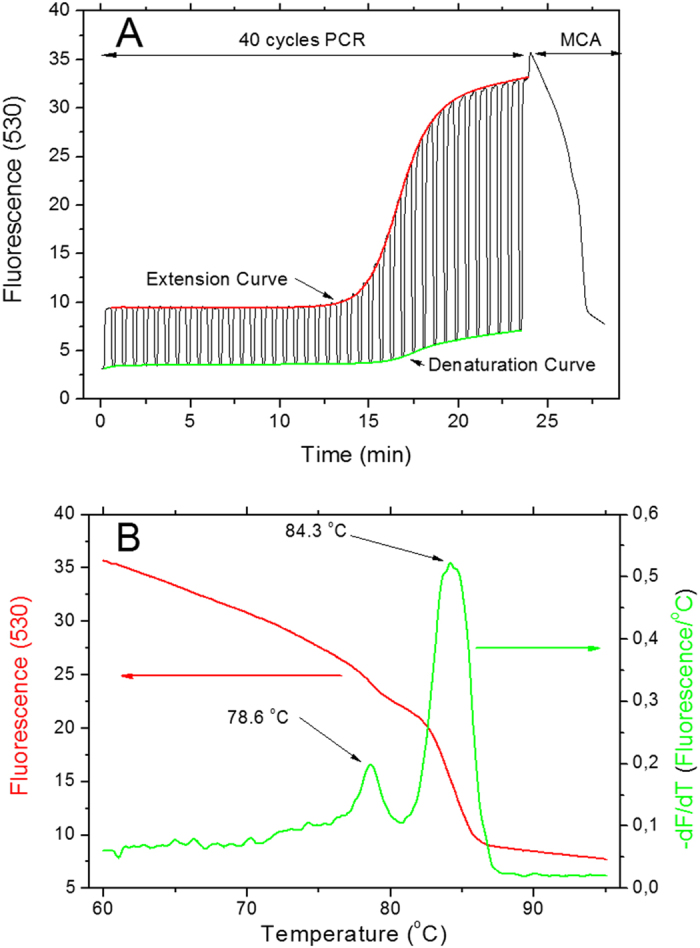
(**A**) A typical data set with both amplification curves extracted from a 40-cycle PCR. The fluorescence amplitude recorded at the end of the extension (red) and denaturation (green) step are shown here. The normalized amplification curves are shown as inset. At the end of the PCR we performed the MCA. (**B**) The results of the MCA analysis showing DNA templates with two different melting temperatures, 78.6 °C and 84.3 °C.

We captured all fluorescent data during the entire PCR protocol (see Fig. 1A) and processed them in Matlab, a numerical computing software. We extracted data from Roche LightCycler in two blocks, temperature as a function of time and fluorescence as a function of time. We then used temperature data to determine the beginning of each cycle. Next, we extracted fluorescence amplitude during denaturation and extension steps for each cycle. Each point was formed as a mean from the last five fluorescence measurements in the step. This data processing resulted in two amplification curve denaturations and extensions as a function of cycle number.

We defined the cycle threshold (C_T_) as a value of the cycle number when fluorescence amplitude is equal to the background mean (from first 10 cycles) plus five times the standard deviation of the average. We calculated the C_T_ values for both curves for denaturation as well as extension. As the last step of data processing, both amplification curves were normalized by subtracting the initial fluorescence signal and then dividing all numbers by the fluorescence amplitude. We also performed MCA (see Fig. 1B).

Concentrations were determined by performing PCR with different template concentrations by creating standard curves.

## Results

**Figure 2:**
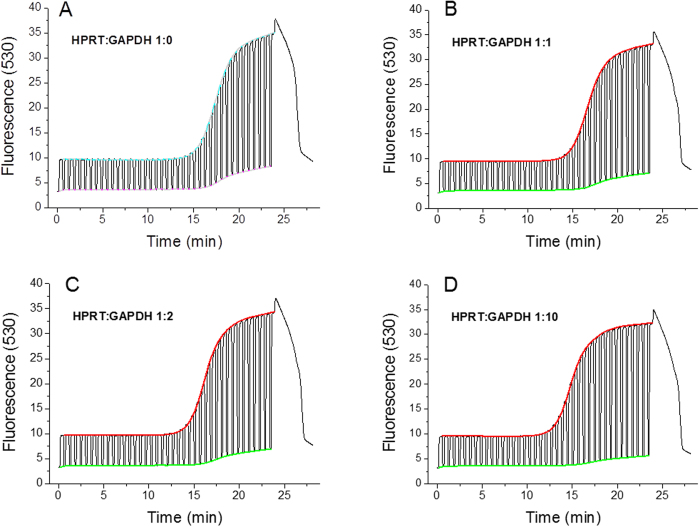
Raw fluorescence data from LightCycler for experiments with different ratios between HPRT and GAPDH genes. (**A**) Shows HPRT gene alone, (**B**) ratio 1:1, (**C**) ratio 1:2 and (**D**) ratio 1:10. We extracted both denaturation (green) and extension (red) curves from all graphs. No template control (NTC) expressed no amplification and its average fluorescence amplitude was 0.113 with a standard deviation of 0.002.

**Figure 3:**
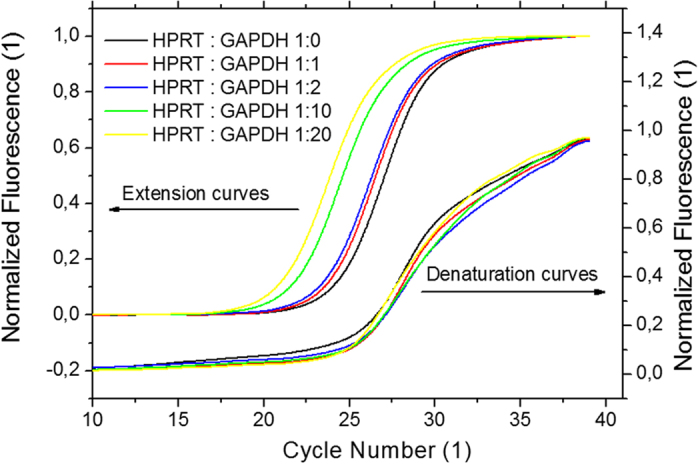
(left axis) Extracted normalized extension curves. The curves’ amplitude was divided by its maximum amplitude and the slope was also subtracted for easier curve to curve comparison. (right axis) The normalized denaturation curves. The normalization was done in a similar fashion as for extension curves.

The captured data from PCR with different DNA template concentrations are shown in process in Fig. 2. Normalized PCR amplification curves are shown in Fig. 3. As an example, we performed an experiment with a constant concentration of HPRT and a varied concentration of GAPDH.

### Non-TaqMan Gene in excess

#### Housekeeping genes

**Figure 4:**
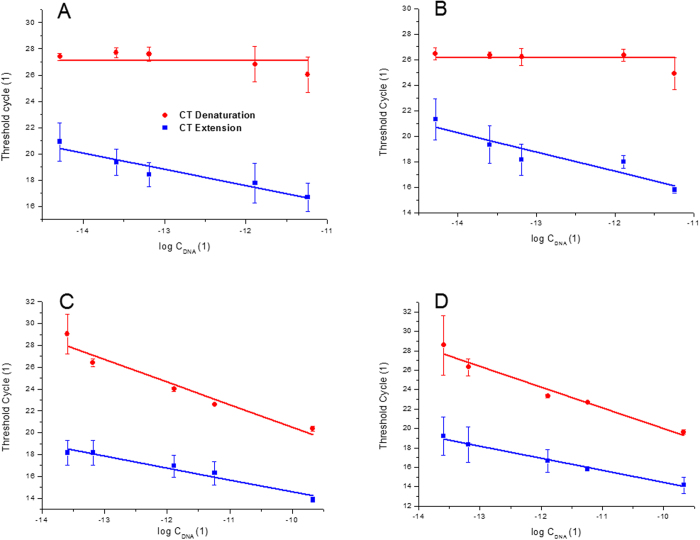
Extracted threshold cycles (C_T_) for experiments with fixed concentration of either HPRT (**A**,**B**), GAPDH (**C**) or HA (**D**) genes and varying concentrations of GAPDH (**A**) HA (**B**) or HPRT (**C**,**D**). Each experiment was repeated three times. The varied concentration was calculated by subtracting the constant value of concentration (6.25 × 10^−7^ ng/μL) from the total DNA concentration. The red circles correspond to CT extracted from the denaturation curves while the blue squares from the extension curves.

First, we kept the TaqMan gene (HPRT) concentration constant while the concentration of GAPDH gene was varied. The C_T_ values extracted from denaturation curves remained constant, as shown in Fig. 4A. The C_T_ values extracted from the extension curves decreased with increasing concentration of the non-TaqMan gene (GAPDH).

#### Viral load

Second, we also kept the TaqMan gene (HPRT) concentration constant while we varied concentration of cDNA of HA gene. The C_T_ values extracted from denaturation curves again remained constant as shown in Fig. 4B and the C_T_ values extracted from the extension curves decreased with increasing concentration of the non-TaqMan gene (HA).

### TaqMan Gene in excess

#### Housekeeping genes

In the two experiments we conducted, the TaqMan gene is in higher concentrations than the non-TaqMan gene. Figure 4C shows the results of experiments using a varied concentration of the HPRT (TaqMan gene) and using a constant concentration of the GAPDH (non-TaqMan gene). The extracted C_T_ values from the denaturation and extension curves both decreases with an increasing HPRT concentration. However, the rate of change of the C_T_ values extracted from the denaturation curve is higher than the one from the extension curve. Quantitative information on both genes can be determined from the difference between both curves at a particular total DNA concentration.

#### Viral load

As for the housekeeping gene, we have varied concentration of HPRT (TaqMan gene) and constant concentration of HA (non-TaqMan gene). The results (see Fig. 4D) are similar to the previous ones.

### Typical determination of DNA template concentrations

A C_T_ value extracted from denaturation curve in the first sample had an amplitude of 26.9. It corresponded only to the concentration of the HPRT gene. A C_T_ value was also extracted from two extension curves, one with primers for the GAPDH gene (C_T_ = 18.1) and one with primers for the HA gene (C_T_ = 17.5). Those three C_T_ values corresponded to a concentration of 6.25 × 10^−7^ for HPRT, 3.49 × 10^−6^ ng/μl for GAPDH and 4.52 × 10^−6^ ng/μl for HA.

The C_T_ values extracted from the second sample gave an amplitude of 26.5 for HPRT, 18.4 for GAPDH and 16.8 for HA. Those C_T_ values corresponded to a concentration of 6.25 × 10^−7^ ng/μl for HPRT, 2.61 × 10^−6^ ng/μl for GAPDH and 7.55 × 10^−6^ ng/μl for HA.

## Discussion

The ability to perform quantitative multiplexed PCR is of particular interest for a number of applications. Typically, it is performed by probe-based PCR with a number of probes using specific dyes and with corresponding fluorescent channels for each dye. Here, we have demonstrated a novel method enabling the simultaneous detection of two DNA templates, using only a single fluorescent channel.

We combined a FAM type of TaqMan probe for the first DNA template with an intercalating dye for the second DNA template. We recorded a continuous fluorescence signal for 40-cycle PCR protocol, followed by MCA. From the fluorescent signal, we extracted two amplification curves. One was at the end of the denaturation step corresponding only to the TaqMan probe and the second one was at the end of the extension step. The second curve consisted of information from both genes. We then subtracted a TaqMan probe-based data from the second curve resulting in only the non-TaqMan template amplification curve. This extraction was conducted by a script in Matlab. The possibility of detecting and quantifying more than two genes was demonstrated by running several experiments with different primers within the same sample.

An easy way of extracting information on the concentration ratio between two or more genes was demonstrated based on the PCR standard curve. The method feasibility was demonstrated for a concentration difference of up to four orders of magnitude. Results with higher concentration differences are most likely hindered by the depletion of the master mix due to the amplification of one gene.

This method can be implemented for DNA template quantification using TaqMan probe-based gene with a known concentration as the internal standard. In such a case, the standard PCR curve would not be required.

Examples for this can be seen in the experiments in which the concentrations of the three genes were determined. The concentration of the TaqMan gene was the same as for the standard curves in the first sample, This and the fact that the all concentrations were within one order of magnitude allowed a relatively accurate determination of the concentrations of all genes. In the second sample, the concentration of the TaqMan gene deviated significantly from the standard curves. Furthermore, the difference in concentrations between the genes was more than two orders of magnitude. Because of this, the estimate of concentrations became less accurate.

The described method might an option to increase the number of detected genes concurrently without expanding the number of fluorescent channels of commercial real-time PCR devices. Even here, we only tested a single tube, and an identical experiment could be done in all 96 wells of a standard plate system to expand into the detection of 192 genes, 384 into 768 and 1536 into 3072. Furthermore, the method could also be further optimized by research into the binding properties of the intercalating dyes.

In this contribution, the C_T_ values were extracted from a continuous fluorescent signal. Identical information can be also extracted from only two measurement points during each amplification cycle, at the end of the denaturation step and at the end of the extension step. This way, practically any commercial real-time PCR system is capable of doubling the throughput by performing the multiplexing described in this contribution.

## Additional Information

**How to cite this article**: Ahrberg, C. D. and Neužil, P. Doubling Throughput of a Real-Time PCR. *Sci. Rep.* **5**, 12595; doi: 10.1038/srep12595 (2015).

## Figures and Tables

**Table 1:** List of primers and their corresponding sequences.

Gene	Direction	Sequence
HPRT	Forward	5′-TGACCTTGATTTATTTTGCATACC-3′
HPRT	Reverse	5′-CGAGCAAGACGTTCAGTCCT-3′
GAPDH	Forward	5′-AGCCACATCGCTCAGACAC-3′
GAPDH	Reverse	5′-GCCAATACGACCAAATCC-3′
HA	Forward	5′-GGGACTCAACAATTATGAAAAGTGAA-3′
HA	Reverse	5′-GGGTGTATATTGTGGAATGGCAT-3′

**Table 2:** Experiments with excess GAPDH and extracted threshold cycles (C_T_).

Experiment Name	Concentration HPRT in ng/μl	Concentration GAPDH in ng/μl	Denaturation C_T_	Extension C_T_
1:0	6.25 × 10^−7^	0	27.4	20.9
1:1	6.25 × 10^−7^	6.25 × 10^−7^	27.7	19.4
1:2	6.25 × 10^−7^	12.5 × 10^−7^	27.6	18.4
1:10	6.25 × 10^−7^	6.25 × 10^−6^	26.8	17.8
1:20	6.25 × 10^−7^	12.5 × 10^−6^	26.0	16.7

**Table 3:** Experiments with excess HA and extracted threshold cycles (C_T_).

Experiment Name	Concentration HPRT in ng/μl	Concentration HA in ng/μl	Denaturation C_T_	Extension C_T_
1:0	6.25 × 10^−7^	0	26.5	21.3
1:1	6.25 × 10^−7^	6.25 × 10^−7^	26.3	19.3
1:2	6.25 × 10^−7^	12.5 × 10^−7^	26.2	18.2
1:10	6.25 × 10^−7^	6.25 × 10^−6^	26.3	17.9
1:20	6.25 × 10^−7^	12.5 × 10^−6^	24.9	15.8

**Table 4:** Experiments with excess HPRT and extracted threshold cycles (C_T_).

Experiment Name	Concentration HPRT in ng/μl	Concentration GAPDH in ng/μl	Denaturation C_T_	Extension C_T_
1:1	6.25 × 10^−7^	6.25 × 10^−7^	29.0	18.2
2:1	12.5 × 10^−7^	6.25 × 10^−7^	26.4	18.2
10:1	6.25 × 10^−6^	6.25 × 10^−7^	24.0	17.0
20:1	12.5 × 10^−6^	6.25 × 10^−7^	22.6	16.3
100:1	6.25 × 10^−5^	6.25 × 10^−7^	20.4	13.9

**Table 5:** Experiments with excess HPRT and extracted threshold cycles (C_T_).

Experiment Name	Concentration HPRT in ng/μl	Concentration HA in ng/μl	Denaturation C_T_	Extension C_T_
1:1	6.25 × 10^−7^	6.25 × 10^−7^	28.6	19.2
2:1	12.5 × 10^−7^	6.25 × 10^−7^	26.3	18.3
10:1	6.25 × 10^−6^	6.25 × 10^−7^	23.3	16.6
20:1	12.5 × 10^−6^	6.25 × 10^−7^	22.7	15.9
100:1	6.25 × 10^−5^	6.25 × 10^−7^	19.6	14.1
